# Diversity of the Amoebozoa and Ciliophora Groups in Non-Human Primates Kept Ex Situ and in Their Handlers in Different Institutions in Brazil

**DOI:** 10.3390/pathogens14010056

**Published:** 2025-01-10

**Authors:** Laís Dib, Breno da Silva, Lais Correa, Alcides Pissinatti, Silvia Moreira, Maria Clotilde Tavares, Rodrigo Teixeira, André Luíz da Costa, José Augusto Muniz, Amauri Junglos, Zelinda Maria Hirano, Aline Dada, Sidnei da Silva, Maria Regina Amendoeira, Alynne Barbosa

**Affiliations:** 1 Laboratory of Protozoology, Oswaldo Cruz Institute, Oswaldo Cruz Foundation, Av. Brasil, 4365, Rio de Janeiro 21040-360, RJ, Brazil; laisverdandib@gmail.com (L.D.); amendoeira.fiocruz@gmail.com (M.R.A.); 2 Campos School of Medicine (FMC), Av. Alberto Torres, 217, Campos dos Goytacazes 28035-581, RJ, Brazil; 3 Laboratory of Parasitology, Fluminense Federal University, Alameda Barros Terra Blvd., no number, Niterói 24020-150, RJ, Brazil; brenotorres@id.uff.br (B.d.S.); laislisboa@id.uff.br (L.C.); 4 Rio de Janeiro Primatology Center—CPRJ/INEA, Paraíso St., no number, Guapimirim 25940-000, RJ, Brazil; alcidespissinatti@gmail.com (A.P.); silviabm.inea@gmail.com (S.M.); 5 Primatology Center, University of Brasília, Park Way, no number, Brasília 71750-000, DF, Brazil; mchtavares@gmail.com; 6 Quinzinho de Barros Municipal Zoo (Zoo Sorocaba), Teodoro Kaisel St., 883, Sorocaba 18020-268, SP, Brazil; rhftzoo@hotmail.com (R.T.); almotacosta@yahoo.com.br (A.L.d.C.); 7 Graduate Program in Wild Animals, Paulista State University “Julio de Mesquita Filho” (UNESP), Av. Prof. Mário Rubens Guimarães Montenegro, no number, Botucatu 18618-687, SP, Brazil; 8 Department of Wildlife Medicine, University of Sorocaba (UNISO), Raposo Tavares Rd., km 92.5, Sorocaba 18023-000, SP, Brazil; 9 National Primate Center, BR-316 Hwy., no number, Ananindeua 67033-009, PA, Brazil; japcmuniz@gmail.com (J.A.M.); amauri.junglos@cenp.gov.br (A.J.); 10 Bugio Project, Regional University of Blumenau, Rio de Janeiro St., 401, Indaial 89086-000, SC, Brazil; zehirano@hotmail.com (Z.M.H.); alinenaissa@gmail.com (A.D.); 11 Evandro Chagas National Institute of Infectious Diseases, Oswaldo Cruz Foundation, Av. Brasil, 4365, Rio de Janeiro 21040-360, RJ, Brazil; sidnei.silva@ini.fiocruz.br

**Keywords:** protists, zoonosis, in vitro isolation, molecular characterization

## Abstract

Parasitic infections in non-human primates (NHPs) kept ex situ can be caused by zoonotic protists like *Balantioides coli* and *Entamoeba histolytica*. In Brazil, little is known about these infections in neotropical species. This study aimed to identify Amoebozoa and Ciliophora groups in fecal samples through in vitro isolation and molecular analysis, mapping their distribution in Brazil. Among 511 NHP and 74 handler’s fecal samples, Amoebozoa were found in 61 (11.9%) NHP samples, and Ciliophora in 6 (1.2%). Amoebic cysts were present in 12 (16.2%) human samples. *Iodamoeba* sp. from *S. xanthosternos*, *E. coli* from a handler, and *B. coli* from *P. troglodytes* and *A. guariba* were isolated in vitro. Molecular techniques identified *E. dispar* (34.2%), *E. histolytica* (5.1%), *E. hartmanni* (26.6%), *E. coli* (15.2%), *Iodamoeba* sp. (12.6%), *E. nana* (8.9%), and *B. coli* (7.6%). Greater protist diversity occurred in northern and southeastern regions, with *E. histolytica* and *B. coli* detected in endangered species, such as *Saguinus bicolor* and *Alouatta guariba*. Protist overlap between humans and NHPs underscores zoonotic risks. This study presents the first molecular characterization of Amoebozoa and Ciliophora in neotropical NHPs kept ex situ in Brazil, highlighting the need for improved hygiene and management protocols in primate institutions.

## 1. Introduction

Parasitic infections in non-human primates (NHPs) kept ex situ, caused by agents like *Balantioides coli* (Malmsten, 1857) and *Entamoeba histolytica* (Schaudinn, 1903), can lead to severe health issues such as dysentery, dehydration, and death in both humans and NHPs [1,2,3]. Other asymptomatic species, such as *Entamoeba dispar* (Brumpt, 1925), *Entamoeba hartmanni* (Prowazek, 1912), *Entamoeba coli* (Grassi, 1879), *Iodamoeba* sp. (Dobell, 1919), and *Endolimax nana* (Wenyon & O’Connor 1917), have similar characteristics to *E. histolytica*. Additionally, species like *Entamoeba moshkovskii* (Calaja, 1941), *Entamoeba nuttalli* (Castellani, 1908), and *Entamoeba polecki* (Prowazek, 1912) can cause specific infections in humans or are frequently found in NHPs [4,5,6,7,8]. Transmission can occur indirectly through contaminated water and food or directly via host contact, especially in ex situ environments where NHPs engage in social grooming, facilitating parasite spread [3,9,10]. Animal handlers can also become infected through the improper handling of contaminated feces, potentially transferring parasites to NHP enclosures and other areas [11,12].

In general, these intestinal protists can only be detected in NHPs and humans by analyzing fecal samples via routine parasitological methods. However, a more detailed taxonomic classification involves the combination of different methods, such as in vitro isolation, molecular tools, and morphological characterization, in order to carry out in-depth analyses of the genetic material, its variants, subtypes, and the morphologies of parasitic forms. Nevertheless, few studies have used a combination of these methods, most of them carried out in the Philippines, Japan, China, and Brazil [13,14,15,16,17,18,19]. In this context, protists of the Amoebozoa and Ciliophora groups have been characterized mostly via microscopic techniques in several countries [1,2,11,12,20,21,22,23,24,25,26].

The study of gastrointestinal parasites in NHPs kept ex situ has significant relevance in public health, as it provides essential insights into the complexity of host–parasite interactions outside their natural environments [27]. Also, this analysis contributes to a better understanding of the potential zoonotic routes in primate institutions due to phylogenetic proximity between NHPs and provides information to improve the management protocols [28]. In this scenario, the examination of fecal samples allows for insight into how parasite life cycles are maintained in artificial conditions and how these organisms adapt to new ecological contexts. Furthermore, monitoring parasites in ex situ primates and their handlers contributes to preserving the genetic diversity of humans, NHPs, and parasites, as these organisms play critical roles in population regulation, ecosystem dynamics, and gut microbiota [29,30].

Although Brazil has one of the greatest diversities of NHPs in the world [31], knowledge about the frequency and classification of intestinal protists is still scant, especially in neotropical animals kept under human care. Given this scenario, the purpose of this study was to characterize the parasites of the Amoebozoa and Ciliophora groups in fecal samples and in vitro isolation using staining and molecular tools, and to determine the distribution of parasitic species in different regions of Brazil.

## 2. Materials and Methods

### 2.1. Study Area and Fecal Samples Collection

Fecal samples from NHPs raised ex situ, their handlers (*n* = 63), veterinarians (*n* = 6), and food handlers (*n* = 5) were collected at five Brazilian institutions according to the protocol carried out in a previous study [32]. In this study, it was decided to sample at least one institution from each of the 5 regions of Brazil in order to obtain the broadest parasitological overview possible, since the number of NHPs in each institution varies significantly. The only region where it was not possible to conduct the parasitological survey was the northeast. Sampling was conducted in all enclosures of the institutions, in addition to fecal collection from all the professionals who worked directly with NHPs and agreed to participate in this study. Fecal samples were collected from the families Callitrichidae (*n* = 225), Aotidae (*n* = 54), Cebidae (*n* = 96), Pitheciidae (*n* = 14), Atelidae (*n* = 95), Cercopithecidae (*n* = 16), Hominidae (*n* = 8), and Lemuridae (*n* = 3) of NHPs (Table 1), and from members of the animal care team (*n* = 74). All the NHP samples were collected in the morning directly from the floor of each enclosure from March 2021 to June 2023, mainly in the summer. Institution A (*n* = 68) is a zoo located in the city of Sorocaba, Southeast region of Brazil (23°30′21″ S and 47°26′ 17″ O); Institution B (*n* = 36) is a primate center located in Brasília University, Central-West region (15°56′53″ S and 47°56′10″ W); Institution C is a Primatology Center built close to native Atlantic Forest in Rio de Janeiro (*n* = 176), also in the Southeast region (22°29′18″ S and 42°54′48″ W); and Institution D (*n* = 206) is a Primate Center focused in animal research, reproduction, and conservation. It is surrounded by Amazonia forest and located in Ananindeua, North region of Brazil (1°23′02″ S and 48°22′ 51″ W); and Institution E (*n* = 25) is a place specialized in the conservation of *Alouatta* located in Indaial, South region (26°53′51″ S and 49°13′36″ W).

### 2.2. Microscopic Parasitological Techniques

Immediately after the fecal samples from the NHPs were collected and those of the handlers were received, all the fecal material was subjected to the direct smear for the detection of trophozoites of the Amoebozoa and Ciliophora groups. This initial analysis was carried out at the facilities of the institutions that keep NHPs, aiming to recover as many positive samples as possible with viable parasitic forms. Subsequently, all the samples were sent to the Parasitology Laboratory of the Federal Fluminense University in Niteroi, RJ, where qualitative techniques were also carried out to identify cystic forms, including centrifugal flotation with zinc sulfate solution (d = 1.180 g/mL) [33], centrifugal sedimentation with modified ethyl acetate [34,35], and spontaneous sedimentation [36]. Reading of the microscopy slides, morphometry, and photomicrography of the parasites’ shapes was carried out using an Olympus BX41 optical microscope (Olympus, Tokio, Japan) and a BEL^®^ EU12CONVS digital camera (BEL^®^, Newcastle, UK) under 40 and 1000× magnification.

### 2.3. In Vitro Protist Cultivation

All the fecal samples that showed motile trophozoites on direct examination and/or cysts were inoculated in 8 mL of culture media in glass test tubes (15 × 1.8 cm) with screw caps containing the xenic media modified Pavlova [37,38] and TYSGM-9 [39]. A total of 60 µL of positive samples were inoculated into each tube in a set of four tubes. Two of these tubes contained modified Pavlova medium, one with horse serum and the other with fetal bovine serum. The other two remaining tubes contained the TYSGM-9 medium with the respective sera. Antibiotic solutions containing streptomycin and penicillin (dilution: 0.3%—1.5 mL of the solution at 10.000 U/µL for each 500 mL of xenic medium) were added to all the media, as well as one drop of rice starch suspension (dilution: 6.25%—1 g of starch in 16 mL of the medium to be used). Once inoculated, each isolate was incubated in a bacteriological incubator at 36 °C, and the sediment in the tube was examined daily at 24 h intervals for seven days. From the 3rd day onwards, all the samples showing viable and motile parasites were considered successfully isolated. The protozoa were maintained in vitro at intervals of 48 h to 72 h of subcultures, i.e., inoculation of the isolate into new and fresh media. Finally, to observe the parasites in detail, trophozoites from the Ciliophora group were subjected to Differential Interference Contrast (DIC) and the isolates from the Amoebozoa group underwent Wheatley Trichrome staining.

### 2.4. Wheatley Trichrome Staining—Amoebozoa Group

A smear was made with 10 µL of the culture sediment on a 22 × 22 mm slide containing 5 µL of Mayer’s albumin. This material was then immersed in Schaudinn’s Fixative (saturated solution of HgCl_2_, 95% alcohol, and glacial acetic acid) for 10 min. Subsequently, the material was transferred to a Petri dish containing 70% iodine ethanol solution for three minutes, followed by another Petri dish containing 70% ethanol for another three minutes. The smear was then submerged in the Trichrome dye solution (0.6% Chromotrope 2R, 0.3% Fast Green, 0.7% phosphotungstic acid, 1% acetic acid, and distilled water q.s.) for 10 min. The next step consisted of briefly passing (3–5 s) the material through the bleaching solution (90% ethanol and 1% glacial acid). After decolorization, the material was dehydrated in absolute ethanol for 2 passes of three minutes each. Lastly, the material was placed in a Petri dish with xylene and left there for five minutes. After clarification, the slide was mounted on a microscope slide using Entellan^®^ synthetic resin (Merck, Darmstadt, Germany) for the subsequent identification and measurement of trophozoites. Parasite cells were examined under an optical microscope to measure the length and width of the trophozoites, as well as the length and width of the nucleus.

### 2.5. Differential Interference Contrast (DIC)—Ciliophora Group

The ciliate culture material was subjected to consecutive washes with a physiological solution at 1500 RPM for five minutes to clean the isolates, thereby removing bacteria and artifacts. After this procedure, a drop of the sediment was deposited between a 22 × 22 mm slide and coverslip, and the protists, still alive, were examined under an optical microscope until the parasite cells became immobile. The parasites were observed under an optical microscope and the parameters analyzed were the length and width of the trophozoite; the length, width, position, and shape of the nucleus; and the number of contractile and food vacuoles.

### 2.6. Molecular Characterization

The fecal samples positive for the Amoebozoa and Ciliophora groups in coproparasitological techniques, as well as the isolates taken directly from the culture, were subjected to DNA extraction using a QIAamp Fast DNA Stool Mini Kit (QIAGEN, Hilden Germany). In order to break the cystic wall and optimize DNA extraction from fecal samples containing only cystic forms, pre-processing was carried out, which consisted of leaving the sample containing lysis buffer for three minutes in liquid nitrogen and then in a dry bath incubator at 95 °C, also for three minutes. This step was repeated three times, and immediately following the last heating cycle, the sample was subjected to the extraction protocol of the kit following the manufacturer’s instructions. 

*Entamoeba histolytica*, *E. dispar*, and *E. moshkovskii* were identified by nested PCR (polymerase chain reaction) using the following protocols: primary PCR with 2.5 µL of buffer 10×, 0.8 µL of 1.5 mM MgCl_2_, 1.5 µL of a 2.5 mM DNTP, 2 µL of each primer (E1 and E2) at 10 pmol, 0.3 µL of Taq polymerase 5U Platinum™ (Invitrogen, São Paulo, Brazil), 0.5 µL of 50 mM BSA, 12.9 µL of ultrapure water, and 2.5 µL of DNA, making a total volume of 25 µL [40]. This same protocol was employed in the secondary PCR reactions of *E. histolytica*, *E. dispar*, and *E. moshkovskii*, but with their respective primers [40,41]. The amplicon used in the secondary PCR was analyzed both without dilution and with a 1:10 dilution. Cycling was 3 min at 95 °C, 40 cycles at 94 °C for 50 s, 50 °C for 90 s and 72 °C for 2 min, and a final extension of 72 °C for 7 min.

The molecular characterization of *E. coli*, *E. polecki*, *E. hartmanni*, *E. nuttalli*, *E. nana*, and *Iodamoeba* was performed by a conventional PCR using the same protocol as the one employed in the primary PCR for *E. histolytica*, *E. dispar*, and *E. moshkovskii*. However, the temperature cycling for each species was different from that described above. The reactions were carried out with an initial denaturation step at 95 °C for seven minutes, followed by 40 cycles at 95 °C for 30 s and 55 °C for 30 s for *E. polecki*, 58 °C for 30 s for *E. nana* and *E. hartmanni*, 60 °C for 30 s for *Iodamoeba* spp., and 62 °C for 30 s for *E. coli*; an extension step at 72 °C for 30 s; and a final extension step at 72 °C for seven minutes. For *E. nuttalli*, a denaturation temperature of 94 °C was applied for 15 s, 60 °C for 30 s, and an extension at 72 °C for 30 s [42,43,44,45].

For the Ciliophora group, primers were used that amplify a fragment of the ITS1–5.8s RNAr-ITS-2 region of the nuclear gene [46]. The protocol was a conventional PCR in which used a total of 25 µL containing 12.5 µL of Platinum™ Hot Start PCR Master Mix (Invitrogen, Itapevi, Brazi) l2.5 µL of each primer (B58D 5′-GCT CCT ACC GAT ACC GG GT-3′ and B58RC 5′-GCG GGT CAT CTT ACT TGA TTT C-3′) at 10 pmol, 2.5 µL of ultrapure water, and 5 µL of DNA. Amplification was performed at 94 °C for 10 min, 31 cycles of 94 °C for 1 min, 60 °C for 1 min, 72 °C for 1 min, and a final extension at 72 °C for five minutes.

Positive and negative controls were used for all the PCR assays. The amplified products were examined in 1.5% agarose gel. Whenever an amplified PCR product came from a potentially pathogenic genus, up to six samples of each product were subjected to purification with the Exo-SAP enzyme and to sequencing in order to confirm the amplification of the groups of protists under study. Forward and reverse sequencing was carried out in a 3730 DNA Analyzer (Applied Biosystems^®^, San Franscisco, CA, USA) on the Fiocruz platform. The ciliate variants detected from the ITS-1 and ITS-2 hypervariable regions was also carried out on samples positive for the Ciliophora group [46].

The sequences were edited using the BioEdit version 7.2.5 software and were compared with each other and with the GenBank references in order to identify similarities between them. This analysis required the comparison of a nucleotide sequence from the NHP samples and from the handler samples for each protist taxon in addition to the pathogenic species *B. coli* and *E. histolytica*.

## 3. Results

### 3.1. Microscopic Analysis and Protists’ In Vitro Isolation

A total of 511 fecal samples were collected from NHPs living ex situ at five Brazilian institutions located in different regions of the country. A microscopic parasitological analysis of the NHP samples revealed the presence of parasitic forms of the Amoebozoa and Ciliophora groups in 61 (11.9%) and 6 (1.2%) samples, respectively. In addition, 74 fecal samples from NHP handlers were also analyzed, with only 12 (16.2%) being positive for cysts of the Amoebozoa group, as described previously [32]. Thus, a total of 79 fecal samples, comprising NHPs and handlers, were positive for these parasite groups.

**Figure 1:**
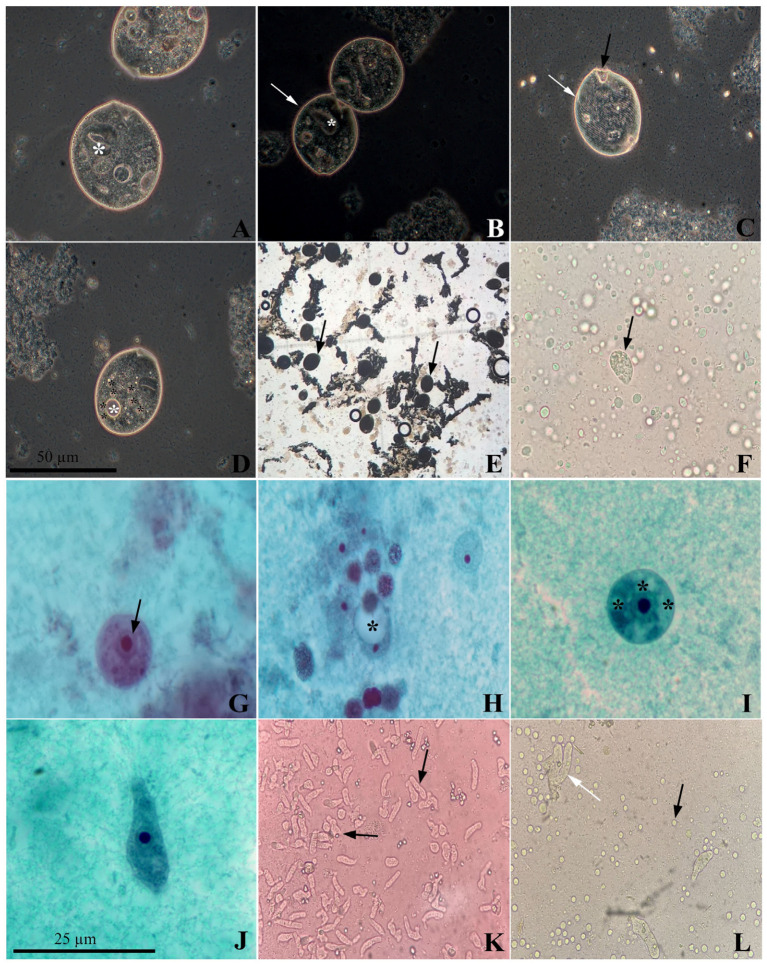
*Balantioides coli* isolate, visualized by Differential Interference Contrast (DIC) microscopy, obtained from a sample of *Pan troglodytes* feces. (**A**) Trophozoite with a kidney-shaped nucleus (*). (**B**) *B. coli* trophozoites with a kidney-shaped nucleus (*) in the process of binary fission with conspicuous cilia (white arrow). (**C**). Trophozoite with striated body surface (white arrow) and cytostome in the anterior region (black arrow). (**D**) Contractile vacuole (* white) and seven food vacuoles (* black). (**E**) *B. coli* trophozoites (black arrow) maintained in vitro from *P*. *troglodytes*. (**F**) *B. coli* trophozoite isolated from a sample of *A. guariba* feces. (**G**) Cystic form of *Iodamoeba* sp. 13 µm in diameter with the membrane surrounding the nucleus (black arrow). (**H**) Cyst with clearly visible glycogen vacuole (*). (**I**) Cyst with 14 µm diameter containing several food vacuoles (*). (**J**) Amoeboid-shaped trophozoite with barely visible glycogen vacuole. (**K**) Trophozoites of *Iodamoeba* sp. (black arrow) maintained in vitro from a fecal sample of *Sapajus xanthosternos*. (**L**) *E. coli* trophozoites isolated in vitro from a handler’s fecal sample (white arrow), showing the proliferation of *Blastocystis* sp. in a modified Pavlova culture medium (black arrow). Source: the authors.

Based on the microscopy results, all the samples positive for the aforementioned groups of protists were inoculated to attempt in vitro isolation in modified Pavlova and TYSGM-9 media. This led to the successful in vitro culture of four isolates from the Amoebozoa group. Of these, two came from the feces of *Alouatta caraya*, one from a handler (Institution D), and one from the feces of *Sapajus xanthosternos* (Institution C). The Ciliophora group was isolated from a sample of Pan troglodytes and another from Alouatta guariba, with the former NHPs kept under human care in São Paulo (Institution A) and the latter in Rio de Janeiro. Only the isolates from *S. xanthosternos* and *P. troglodytes* remained viable for more than three days and showed an abundance of parasite cells in the modified Pavlova medium mixed with horse serum. Using molecular analysis and the observation of the parasites in Trichrome staining and DIC, these isolates were later identified as *Iodamoeba* sp. and *Balantioides coli*, respectively (Figure 1).

### 3.2. Protists’ Molecular Characterization and Distribution in Brazil

A total of seven taxa were detected upon associating the analysis of feces and cultures from NHPs and their handlers: *E. dispar* 34.2% (27/79), *E. histolytica* 5.1% (4/79), *E. hartmanni* 26.6% (21/79), *E. coli* 15.2% (12/79), *Iodamoeba* sp. 12.6% (10/79), *E. nana* 8.9% (7/79), and *B. coli* 7.6% (6/79) (Table 2). The most frequent amoeba species were *E. dispar*, which was detected mainly in Aotus infulatus, and *E. hartmanni*, which was more prevalent in Old World NHPs such as *P. troglodytes* and *Chlorocebus aethiops*. On the other hand, the least frequent species in this study was *E. histolytica*, which was detected only in neotropical primates (Table 2). As for the Ciliophora group, samples from both New World and Old World NHPs were positive, with emphasis on *S. libidinosus*, which showed the highest positivity (Table 2).

Cases of coinfection were observed in six samples from *A. infulatus*: *E. dispar* + *E. hartmanni* + *E. coli* (2); *E. dispar* + *Iodamoeba* sp. (1); *E. dispar* + *Iodamoeba* sp. + *E. hartmanni* (1); *E. dispar* + *E. hartmanni* (1); and *E. dispar* + *Iodamoeba* sp. + *E. hartmanni* + *E. coli* (1). In addition, two cases of coinfections were found in the feces from *Alouatta caraya*, one by *E. dispar* + *E. hartmanni* + *E. coli* + *E. nana* and the other by *E. coli* + *E. nana*. A similar situation was detected in *C. aethiops*, which presented polyparasitism in five samples: *E. dispar* + *E. hartmanni* (1); *E. hartmanni* + *E. nana* (2); *E. dispar* + *E. hartmanni* + *E. nana* (1); and *E. dispar* + *E. hartmanni* + *E. coli* (1). Another case of coinfection was detected in a fecal sample from *A. guariba* that tested positive for *E. histolytica* + *B*. *coli*.

The species *E. dispar*, *E. hartmanni*, and *E. coli* were detected in the fecal samples from both NHPs and handlers (Table 3). Percent identity ranging from 96.65% to 100% was found between the gene sequences of *E. dispar* and *E. hartmanni* when comparing the DNA of the parasite amplified from simians and their caretakers with the reference sequences deposited in GenBank. However, the nucleotide sequences of *E. coli* analyzed from the handlers and NHPs in this study showed a lower percent identity (86.38%). Nevertheless, when compared with reference sequences from related hosts, the percent identity values were higher (98.02% and 99.63%) (Table 3). In addition to these species, the same percent similarity (99.67%) was observed between the fragment of *E. histolytica* from *A. guariba* in this study and the reference sequences of *E. histolytica* detected in humans and *Macaca fascicularis* (Raffles, 1821) deposited in GenBank (Table 3).

*Balantioides coli* ITS-1 and ITS-2 hypervariable regions were analyzed to determine the variants of this protist (types A or B). It was found that five presented *B. coli* variant B0, while only the isolate from *P. troglodytes* was variant A0. The latter variant showed an identity greater than 99% when compared with the *B. coli* sequence detected in humans from GenBank (Table 3). The sequences were deposited in GenBank under accession numbers PP761305-PP761310 for *B. coli* and PP769370-PP769382 for *Entamoeba* species.

**Figure 2:**
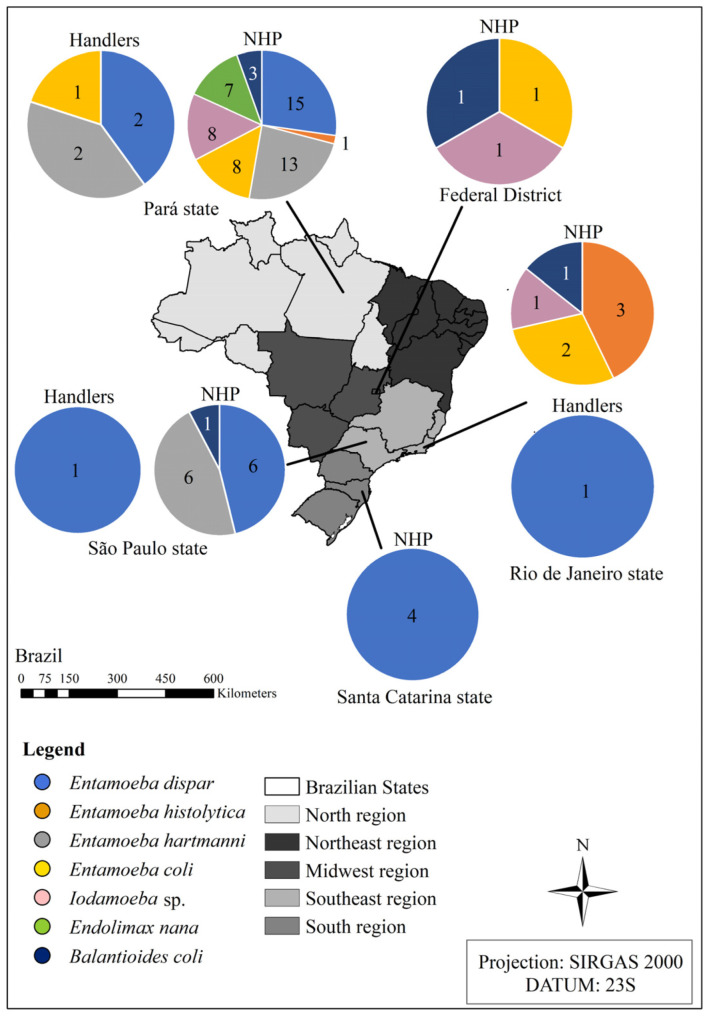
Diversity of species from the Amoebozoa and Ciliophora groups detected in the fecal samples from the non-human primates kept ex situ and from their handlers located in different institutions in Brazil in the states of Pará, Rio de Janeiro, São Paulo, Santa Catarina, and the Federal District. Source: the authors.

After classifying the parasites of the Amoebozoa and Ciliophora groups, it was found that the greatest diversity of parasite taxa was concentrated in the northern region and Rio southeast region, while the institution with the lowest variety of species was the one located in southern Brazil (Figure 2).

## 4. Discussion

Microscopy techniques are very insensitive methods for detecting cysts and trophozoites in feces and they do not enable the specific identification of protists with very similar or even identical morphology, such as *E. histolytica*/*E. dispar*/*E. nuttalli*/*E. moshkovskii* and *B. coli*/*Buxtonella* sp. (Jameson, 1926), which can infect NHPs [4,6,7]. In view of the above, the identification of species considered pathogenic for simians, such as *E. histolytica*, *E. nuttalli*, and *B. coli*, is of utmost importance, as it precludes unnecessary antiparasitic treatments in the cases of infections with non-pathogenic species. Therefore, it minimizes the possibility of forming a generation of protists resistant to the drugs routinely administered to treat amoebiasis and balantidiasis, such as metronidazole [5,10,47]. Moreover, it prevents the excessive metabolization of drugs and consequent kidney and liver damage to animals, thus favoring animal well-being. Unfortunately, microscopic techniques still are the methods most used in routine diagnostics in primate institutions in Brazil due to their low cost and lack of financial investment by the government.

Among the species identified by molecular analysis, the most frequently detected in NHPs was *E. dispar*, except for Institution B in Brazil’s Central-West region. In general, *E. dispar* has been one of the species of the genus *Entamoeba* most frequently detected in the parasitological surveys of feces from Old World NHPs carried out in the Philippines and China [18,48]. Furthermore, *E. dispar* has already been detected as the only species of the *E. histolytica*–*E. dispar* complex infecting different species of Old World and New World NHPs in Japan [49]. In general, the findings of this study were already expected, given that in Brazil, among the species of the *E. histolytica*–*E. dispar* complex, the latter has been the most frequently diagnosed in human samples via molecular techniques [50].

Although most of the NHPs in this study were asymptomatic and did not have loose feces during collection, two individuals of *A. caraya* admitted to the veterinary clinic were receiving specific treatment for amoebiasis during the period of sample collection at the institution located in Pará. One of the isolates from the Amoebozoa group was obtained from one of these individuals, and was characterized as *E. dispar*. It is known that *E. dispar* is still considered non-pathogenic, and that the host is an asymptomatic carrier. However, one case of symptomatic infection in humans has already been reported in an Italian patient [51]. Given this possibility and the lack of information about infection by these protists in NHPs, especially in neotropical species, more clinical studies are needed to determine the real pathogenic potential of these species in different taxa of NHPs.

In addition to *E. dispar*, other species considered non-pathogenic were also detected in NHP feces, including *E. hartmanni* in institutions in Pará and São Paulo and *E. coli* in animals under human care in animal care facilities in Pará, Distrito Federal, and Rio de Janeiro. The diagnosis of these species is in line with what has been reported previously in care facilities for NHPs in Belgium, the Netherlands, Italy, China, and Egypt [12,15,52,53]. In this study, *E. dispar*, *E. hartmanni*, and *E. coli* were also detected in the fecal samples from the NHP handlers, which highlights the potential for zoonotic transmission of these agents. Although these species are not considered pathogenic, they act as bioindicators of fecal contamination and have the same transmission route as pathogenic species. Hence, the case records of these species serve as a wake-up call for the need for improvements in the collective health management of these animals, as well as the individual conduct of the professionals who handle them.

Among the species of the Amoebozoa group considered pathogenic for NHPs, only *E. histolytica* was detected in this study. This protist was identified in the feces of *L. chrysomelas*, *S. bicolor*, and *A. guariba* in Rio de Janeiro and also in a Cebidae NHP in Pará. It is important to highlight that the research team suspects that the location where the vegetables used in the diet of the NHPs at Institution C are sourced may be related to the incidence of *E. histolytica* in these animals, as this region has significant rates of potential human amebiasis cases. In general, *E. histolytica* is reported mainly in Old World NHPs, as indicated in studies conducted in France, the Philippines, China, Belgium, the Netherlands, Germany, and the United Kingdom [18,48,53,54,55,56]. Like in the present study, *E. histolytica* has also been detected in the feces of neotropical NHPs such as *L. chrysomelas*, *Ateles belzebuth* (Geoffroy, 1806), and *Saguinus oedipus* (Linnaeus, 1758) kept under human care in France [56].

The clinical manifestations caused by *E. histolytica* in NHPs are similar to those reported in humans, especially in the case of monogastric NHPs, i.e., which have a single stomach, in which the lesions are more recurrent in the cecum and colon [56,57,58]. In these cases, greenish or brownish watery diarrhea or dysentery are characteristic of the infection [59]. However, in the “leaf-eating lifestyle” NHPs with a multi-compartment stomach, the most frequent cases are of anorexia, lethargy, depression, and diarrhea with no mucus or blood [57,58]. Cases of intestinal amebiasis in *Alouatta belzebuth* (Linnaeus, 1766), *Colobus guereza* (Rueppell, 1835), and *Mandrillus sphinx* with diarrhea were reported in France and extraintestinal conditions in *Semnopithecus entellus* (Dufresne, 1797) and *Colobus guereza* with multifocal and coalescent hepatic nodules have already been mentioned in a study in Germany [55,56]. Although the NHPs in our study showed no clinical changes during the collection period, the presence of *E. histolytica* in monogastric species, including *L. chrysomelas*, *S. bicolor*, and members of the family Cebidae, as well as herbivorous animals with more complex stomachs such as *A. guariba*, must be considered carefully, since these animals will not always present with classic clinical signs of intestinal amebiasis.

Other species such as *E. nuttalli*, *E. moshkovskii*, and *E. polecki* were also included in the panel of this parasitological survey, but they were not detected. It is worth noting that these species have already been reported in other studies with NHPs [6,15,17,53]. It is important to highlight that monitoring *E. nuttalli* in NHP care facilities, as is performed for *E. histolytica*, is advisable, since it is morphologically similar to *E. histolytica* and considered a virulent species causing intestinal and extraintestinal amoebiasis with liver involvement [6,17]. Furthermore, an asymptomatic case of human infection by *E. nuttalli* has also been reported in a zookeeper, underscoring its zoonotic potential [6].

In addition to *Entamoeba*, *E. nana* and *Iodamoeba* sp. were detected in the fecal samples using molecular tools, and *Iodamoeba* sp. was also isolated in vitro from *S. xanthosternos* feces. The diagnosis of these parasite taxa was expected, as they have already been reported in previous studies with NHPs [1,25,26,49,56]. However, most of these studies relied solely on microscopic techniques, likely underestimating prevalence rates. Also, the non-pathogenic *E. nana* and *Iodamoeba* sp. can be mistaken for *Entamoeba* species, making molecular diagnosis crucial, since two *Iodamoeba* strains were identified in humans and NHPs by molecular techniques, underscoring their zoonotic potential [42].

The detection of *E. histolytica* and other amoebae in primates, especially those threatened with extinction, has several public health implications, including potential zoonosis routes and the lack of environmental safety. This is particularly concerning given the possibility that these animals may serve as alternative reservoirs for these protists, as most of them were asymptomatic during this study. It is important to highlight that although the sample size was small, these findings should be considered in future surveys.

With regard to the Ciliophora group, of the six positive samples, two showed trophozoites under direct examination. Using molecular tools, these six samples were confirmed as belonging to *B. coli*, which has been reported in several earlier studies on Old World NHPs [1,7,11,20,22,23,24,26,60,61,62]. However, no other studies reported confirmed *B. coli* infection in New World NHPs and used molecular methods to characterize its variants accurately.

As in the *P. troglodytes* sample, variant A of *B. coli* was predominant in Old World NHP samples housed in European zoos and African sanctuaries, as well as in NHPs kept at a scientific research institution in Brazil [7,14]. Among the attempts at isolation, only the protists identified in the feces of *P. troglodytes* were isolated and maintained in vitro for more than three days, since a large number of trophozoites per field of microscopy was detected only in this case. This abundance of forms of *B. coli* has also been reported in the feces of chimpanzees living in animal care facilities in Japan [24]. It should be noted that variant A0 of *B. coli* has also been reported in human samples from Bolivia [46].

With regard to NHPs, especially Old World ones, *B. coli* infections are commonly reported. In this context, *B. coli* has already been reported as causing cases of diarrhea, dysentery, abdominal distension, anorexia, fever, and dehydration in *Papio hamadryas* kept in an establishment in Saudi Arabia [63]. Other symptomatic cases resulting from infections caused by this ciliate were described in *P. troglodytes* housed at an institution in the United States and in *Gorilla gorilla* reared ex situ in the Republic of Cameroon. In both cases, the animals presented with severe diarrhea, abdominal discomfort, and significant ulcerated lesions in the colon and cecum resulting from a chronic inflammatory reaction. Both the *P. troglodytes* and *G. gorilla* died [64,65]. In addition to affecting the gastrointestinal system, this ciliate has also been identified as one of the parasites provoking the reduction in the fat content in milk in *M. mulatta* in an animal care facility in Puerto Rico, which impairs the development of lactating animals [22]. Although *B. coli* is considered a parasite with zoonotic potential, it was not detected in the fecal samples from the animal handlers in our study.

The detection of pathogenic protists in ex situ environments can lead to infections in other hosts and outbreaks with varying degrees of morbidity and mortality, affecting both NHPs and other animals, particularly in the case of zoos. This situation becomes more critical as it necessitates the precise identification of the agent and the adoption of more complex sanitary strategies, including the isolation and treatment of affected individuals, as well as stricter water and food treatment protocols.

Different species of *Entamoeba* considered non-pathogenic, *Iodamoeba* sp., *E. nana*, and *B. coli* were detected in the feces collected in the same NHP enclosure. This diagnosis may be attributed to cases of coinfections or the fact that in the vast majority of cases, these enclosures house more than one individual due to the behavioral characteristics of primates, which are gregarious animals. It is important to point out that species with a foliaceous and frugivorous diet, such as *Aotus* and *Alouatta*, were the ones with the highest parasite load. This fact may be ascribed to the diet of these animals, as well as their susceptibility to infection by protists, such as the Amoebozoa and Ciliophora groups, since leaf components have anti-helminthic effects. This finding was corroborated by the case of *A. guariba* at an institution in Rio de Janeiro, whose feces was concomitantly positive for *E. histolytica* and *B. coli*.

Cases of polyparasitism, particularly involving pathogenic and commensal protists, highlight the complexity of parasitic interactions in primate institutions. This leads to intricate transmission dynamics making it more challenging to predict and control outbreaks, as parasites could impact the survival and spread of other organisms or hosts. This first molecular survey in NHPs in Brazil associated with microscopic and staining techniques demonstrated that the regular fecal monitoring and molecular characterization of parasitic isolates were essential for detecting potential emerging threats, contributing to NHP conservation and specific interventions.

A comparison of the nucleotide sequences of the protists from the fecal samples of NHPs and their handlers revealed different percent identities, especially in relation to *E. coli* from *A. infulatus* which revealed lower percent identities when compared to human *E. coli*. These percent identities may be attributed to the different subtypes of this protozoa that have been identified in Brazilian biomes [66]. Additionally, the previously discussed homology of *E. coli* indicates that the strains originating from NHPs and their handlers are genetically distinct, which suggests that zoonotic transmission is not occurring in this situation. Furthermore, the short DNA sequence of 18S rRNA is recognized for its high conservation, particularly in the cases of *E. histolytica* and *E. dispar*. Thus, even when 100% homology is observed, it cannot be assumed that the same strain is present in both human and animal hosts. To accurately assess the risk of zoonotic transmission in this scenario, it is essential to conduct analyses targeting polymorphic regions, such as the serine-rich *E. histolytica* protein (SREHP).

In general, the highest diversity and frequency of species from the Amoebozoa and Ciliophora groups was identified in institutions located in Pará, followed by Rio de Janeiro. One of the factors that may also have contributed to the presence of these parasitic agents is the hot and humid climate in the regions, which favors the longer viability of infective cystic forms in the enclosures. These environmental conditions are present in both the Amazon and Atlantic Forest biomes, where the Institutions of Pará and Rio de Janeiro, respectively, are located. Although Brazil is a country of continental dimensions and boasts one of the greatest diversities of non-human primates in the world, it was not possible to obtain a proportionally representative prevalence of positive samples for the Amoebozoa and Ciliophora groups. This favorable scenario for animal conservation in institutions reflects the commitment of professionals to implementing complex and routine personal and collective hygiene practices within the facilities. Nevertheless, similar taxa were detected between NHPs and their handlers, reinforcing the need to refine management protocols, provide continuous training for staff who work directly with NHPs, conduct regular stool examinations for handlers, and monitor the quality of water and food provided to non-human primates, as these are the main sources of infection for these gastrointestinal protists. Furthermore, the logistics for the adapted implementation of laboratory infrastructure necessary for processing fecal samples, isolating, and maintaining protists in the institutions also posed a challenge for the analysis of the samples.

## 5. Conclusions

Followed by the high diversity of non-human primates originating and maintained in Brazil, a wide variety of protist species with zoonotic potential were observed. These agents were dispersed across different regions of the country, even with varied environmental and climatic conditions among states as well as the specific management protocols of each institution. In this scenario, it is possible to observe the versatility and ability of protists to maintain their life cycle in ex situ environments. In this study, the detection of parasitic forms through coprological examinations, combined with in vitro isolation and morphological analysis, proved to be valuable methodologies for the early diagnosis of active infections, especially in asymptomatic animals, thus contributing to a better quality of life for the collection through more detailed monitoring and correct therapeutic measures in the enclosures.

The molecular detection of *Entamoeba*, *Iodamoeba*, *Endolimax*, and *Balantioides* was essential to obtain more precise information about the infection dynamics of these parasites in neotropical non-human primates, which are still extremely under-researched. The high parasitic genetic similarity observed in different NHP hosts under human care indicates its potential zoonotic nature and vulnerabilities during the handling activities of the animals and their waste that need to be adjusted. This situation is a particularly aggravating factor for species under threat of extinction, as observed in this study through the molecular identification of *E. histolytica* in *S. bicolor*, *L. chrysomelas*, and *A. guariba* and of *B. coli* in *A. guariba* and *S. libidinosus*. Clearly, further and more in-depth studies on this topic are needed to shed light on the parasitic agents circulating in Brazil, given that this is only the first study that involved the molecular characterization of species from the Amoebozoa and Ciliophora groups in neotropical NHP species kept under human care in Brazil. This emphasizes the need for routine parasitological monitoring in animal care facilities in Brazil, including samples from animals and humans, using diagnostic tools of high sensitivity and specificity to accurately identify parasitic agents. In addition to that, there is a need to conduct training for the team to improve and even implement new hygiene protocols in the primate institutions and also provide more individual and collective protective equipment, thereby allowing for prophylactic interventions in the parasites transmission routes and thus contributing to the preservation of national biodiversity.

## Figures and Tables

**Table 1:** Fecal samples collected from non-human primates kept ex situ in five different Brazilian institutions.

Non-Human Primates	Samples
Callitrichidae	
*Callithrix* sp. (Erxleben, 1777)	19
*Callithrix aurita* (Geoffroy, 1812)	16
*Callithrix geoffroyi* (Humboldt, 1812)	1
*Callithrix jacchus* (Linnaeus, 1758)	7
*Callithrix penicillata* (Geoffroy, 1812)	19
*Cebuella pygmaea* (Spix, 1823)	2
*Mico argentatus* (Linnaeus, 1771)	1
*Mico chrysoleucus* (Wagner, 1842)	1
*Mico humeralifer* (Geoffroy, 1812)	1
*Mico mauesi* (Mittermeier, Schwarz & Ayres, 1992)	1
*Mico melanurus* (Geoffroy, 1812)	1
*Saguinus bicolor* (Spix, 1823)	11
*Saguinus fuscicollis* (Spix, 1823)	1
*Saguinus martinsi* (Thomas, 1912)	2
*Saguinus midas* (Linnaeus, 1758)	5
*Saguinus niger* (Geoffroy, 1803)	1
*Saguinus ursulus* (Hoffmannsegg, 1807)	8
*Leontocebus weddelli* (Deville, 1849)	11
*Leontopithecus* sp. (Lesson 1820)	1
*Leontopithecus chrysomelas* (Kuhl, 1820)	99
*Leontopithecus chrysopygus* (Mikan, 1823)	4
*Leontopithecus rosalia* (Linnaeus, 1766)	9
*Callimico goeldii* (Thomas, 1904)	4
Aotidae	
*Aotus* sp. (Illiger, 1811)	2
*Aotus infulatus* (Kuhl, 1820)	48
*Aotus nigriceps* (Dollman, 1909)	1
*Aotus trivirgatus* (Humboldt, 1811)	3
Cebidae	
*Cebus albifrons* (Humboldt, 1812)	3
*Cebus kaapori* (Queiroz, 1992)	1
*Cebus olivaceus* (Schomburgk, 1848)	5
*Sapajus* sp. (Kerr, 1792)	2
*Sapajus apella* (Linnaeus, 1758)	22
*Sapajus libidinosus* (Spix, 1823)	15
*Sapajus nigritus* (Goldfuss, 1809)	2
*Sapajus xanthosternos* (Wied-Neuwied, 1826)	12
*Sapajus robustus* (Kuhl, 1820)	3
*Saimiri boliviensis* (Geoffroy & Blainville, 1834)	1
*Saimiri collinsi* (Osgood, 1916)	30
Pitheciidae	
*Callicebus melanochir* (Wied-Neuwied, 1820)	1
*Plecturocebus caquetensis* (Defler, Bueno & García, 2010)	1
*Plecturocebus hoffmannsi* (Thomas, 1908) and *Cheracebus purinus* (Thomas, 1927)	2
*Plecturocebus dubius* (Hershkovitz, 1988)	1
*Plecturocebus vieirai* (Gualda-Barros, Nascimento & Amaral, 2012)	4
*Pithecia mittermeieri* (Marsh, 2014)	1
*Pithecia monachus* (Geoffroy, 1812)	1
*Chiropotes satanas* (*Hoffmannsegg*, *1807*)	1
*Chiropotes utahickae* (Hershkovitz, 1985)	1
*Cacajao melanocephalus* (Humboldt, 1812)	1
Atelidae	
*Alouatta* sp. (Lacepede, 1799)	20
*Alouatta caraya* (Humboldt, 1812)	28
*Alouatta discolor* (Spix, 1823)	1
*Alouatta guariba* (Humboldt, 1812)	33
*Ateles chamek* (Humboldt, 1812)	3
*Ateles paniscus* (Linnaeus, 1758)	2
*Ateles marginatus* (Geoffroy, 1809)	7
*Brachyteles arachnoides* (Geoffroy, 1806)	1
Cercopithecidae	
*Mandrillus sphinx* (Linnaeus, 1758)	1
*Papio hamadryas* (Linnaeus, 1758)	4
*Chlorocebus aethiops* (Linnaeus, 1758)	11
Hominidae	
*Pan troglodytes* (Linnaeus, 1758)	8
Lemuridae	
*Lemur catta* (Linnaeus, 1758)	3

**Table 2:** Molecular characterization of the Amoebozoa and Ciliophora groups using the fecal samples from the New World and Old World non-human primates kept ex situ in five institutions in Brazil.

Host	Positive Samples for Amoebozoa Subjected to Isolation	Positive Samples for Ciliophora Subjected to Isolation	Molecular Identification									NI **
			Feces						In Vitro Cultivation	Feces	In Vitro Cultivation	
			*E. dispar*	*E. histolytica*	*E. hartmanni*	*E. coli*	*Iodamoeba* sp.	*E. nana*	*Iodamoeba* sp.	*B. coli*	*B. coli*	
Callitrichidae												
*Callithrix penicillata*	2	*-*	-	-	-	-	1	-	-	-	-	1
*Callithrix jacchus*	1	*-*	-	-	-	-	1	-	-	-	-	-
*Callithrix humeralifer* and *Leontopithecus chrysomelas*	1	*-*	-	-	-	-	-	-	-	-	-	1
Hybrid *C. jacchus* × *C. penicillata*	-	1	-	-	-	-	-	-	-	1	-	-
*Leontopithecus chrysomelas*	1	*-*	-	1	-	-	-	-	-	-	-	-
*Saguinus bicolor*	2	*-*	-	1	-	-	-	-	-	-	-	1
*Saguinus ursulus*	1	*-*	-	-	-	-	-	-	-	-	-	1
*Saguinus midas* and *Callicebus* sp.*	1	1	-	-	-	1	-	1	-	1	-	-
*Leontocebus weddelli*	1	*-*	-	-	-	-	-	-	-	-	-	1
*Callimico goeldii*	1	-	-	-	-	-	-	-	-	-	-	1
Aotidae												
*Aotus trivirgatus*	1	-	-	-	-	-	-	-	-	-	-	1
*Aotus infulatus* *	12	-	8	-	5	3	5	1	-	-	-	2
Cebidae												
*Saimiri collinsi*	3	-	-	-	1	-	-	-	-	-	-	2
*Cebus olivaceus*	1	-	-	-	-	-	-	-	-	-	-	1
*Sapajus libidinosus* *	1	2	-	-	-	1	1	-	-	2	-	-
*Sapajus xanthosternos*	1	-	-	-	-	-	-	-	1	-	-	-
*Sapajus apella*, *Sapajus libidinosus* and *Cebus albifrons*	1	-	-	1	-	-	-	-	-	-	-	-
Pitheciidae			-									
*Pithecia mittermeieri*	1	-	-	-	-	1	-	-	-	-	-	-
*Cheracebus purinus* and *Plecturocebus hoffmannsi*	1	-	-	-	-	-	-	-	-	-	-	1
Atelidae												
*Alouatta caraya* *	7	-	4	-	1	2	1	2	-	-	-	1
*Alouatta guariba* *	8	1	4	1	-	1	-	-	-	1	-	1
Cercopithecidae												
*Chlorocebus aethiops* *	7	*-*	3	-	6	2	-	3	-	-	-	-
Hominidae												
*Pan troglodytes* *	6	1	6	-	6	-	-	-	-	-	1	-
*Homo sapiens*	12	-	2	-	2	1	-	-	-	-	-	7
Total	73	6	27	4	21	12	9	7	1	5	1	22

*: Coinfection; **: not identified.

**Table 3:** Gene similarity between nucleotide fragments from *E. dispar* (141 to 197 bp), *E. hartmanni* (171 to 178 bp), *E. coli* (303 to 539 bp), *E. histolytica* (380 bp), and *B. coli* (497 bp) detected in the feces of both humans and non-human primates kept under human care.

Sequences	This Study								
	*E. dispar* (*A. caraya*)	*E. dispar* handler	*E. hartmanni* (*C. aethiops*)	*E. hartmanni* handler	*E. coli* (*A. infulatus*)	*E. coli* handler	*E. histolytica* (*A. guariba*)	*B. coli* A0 (*P*. *troglodytes*)	*B. coli* B0 (*C. jacchus* × *C. penicillata*)
*E. dispar* handler (this study)	100%	-	-	-	-	-	-	-	-
*E. dispar* human (KP722600)	100%	100%	-	-	-	-	-	-	-
*E. dispar—Macaca mulatta* (Zimmermann, 1780) (AB282661)	100%	100%	-	-	-	-	-	-	-
*E. hartmanni* handler (this study)	-	-	97.66%	-	-	-	-	-	-
*E. hartmanni* human (MG925066)	-	-	97.71%	100%	-	-	-	-	-
*E. hartmanni—Macaca fascicularis* (PP490721)	-	-	96.65%	100%	-	-	-	-	-
*E. coli* handler (this study)	-	-	-	-	86.38%	-	-	-	-
*E. coli* human (MG925062)	-	-	-	-	86.38%	99.63%	-	-	-
*E. coli—Gorilla gorilla* (Savage, 1847) (AB444953)	-	-	-	-	98.02%	85.29%	-	-	-
*E. histolytica* human (KP233840)	-	-	-	-	-	-	100%	-	-
*E. histolytica*—*Macaca fascicularis* (GQ423749)	-	-	-	-	-	-	100%	-	-
*B. coli* variant A0—human (JF444758)	-	-	-	-	-	-	-	99.74%	92.73%
*B coli* variant A1—*Gorilla gorilla* (JF444760)	-	-	-	-	-	-	-	98.18%	93.07%
*B coli* variant B1—*Gorilla gorilla* (JF444761)	-	-	-	-	-	-	-	91.88%	99.08%

## Data Availability

The nucleotide sequences were deposited in GenBank under accession numbers PP769370 to PP769382 for *Entamoeba* species and PP761305 to PP761310 for *B. coli*.
